# An Enhanced Misinformation Detection Model Based on an Improved Beluga Whale Optimization Algorithm and Cross-Modal Feature Fusion

**DOI:** 10.3390/biomimetics10030128

**Published:** 2025-02-20

**Authors:** Guangyu Mu, Xiaoqing Ju, Hongduo Yan, Jiaxue Li, He Gao, Xiurong Li

**Affiliations:** 1 School of Management Science and Information Engineering, Jilin University of Finance and Economics, Changchun 130117, China; guangyumu@jlufe.edu.cn (G.M.); 6231193021@s.jlufe.edu.cn (X.J.); 6221192039@s.jlufe.edu.cn (J.L.);; 2 Key Laboratory of Financial Technology of Jilin Province, Changchun 130117, China; 3 Changchun Administration Institute, Changchun 130103, China; 4 Faculty of Information Technology, Beijing University of Technology, Beijing 100124, China

**Keywords:** beluga whale algorithm, cross-modal feature, multimodal fusion, misinformation detection

## Abstract

The proliferation of multimodal misinformation on social media has become a critical concern. Although detection methods have advanced, feature representation and cross-modal semantic alignment challenges continue to hinder the effective use of multimodal data. Therefore, this paper proposes an IBWO-CASC detection model that integrates an improved Beluga Whale Optimization algorithm with cross-modal attention feature fusion. Firstly, the Beluga Whale Optimization algorithm is enhanced by combining adaptive search mechanisms with batch parallel strategies in the feature space. Secondly, a feature alignment method is designed based on supervised contrastive learning to establish semantic consistency. Then, the model incorporates a Cross-modal Attention Promotion mechanism and global–local interaction learning pattern. Finally, a multi-task learning framework is built based on classification and contrastive objectives. The empirical analysis shows that the proposed IBWO-CASC model achieves a detection accuracy of 97.41% on our self-constructed multimodal misinformation dataset. Compared with the average accuracy of the existing six baseline models, the accuracy of this model is improved by 4.09%. Additionally, it demonstrates enhanced robustness in handling complex multimodal scenarios.

## 1. Introduction

Online social networks have fundamentally transformed traditional communication patterns, emerging as the primary platform for information sharing and dissemination [1,2,3]. The 54th Statistical Report on Internet Development in China released by CNNIC in 2024 reveals that the number of Chinese Internet users has reached 1.1 billion [4]. However, the rapid expansion of the Internet, combined with inadequate regulation, has created problems for the spread of misinformation [5,6]. Cybercriminals exploit these channels, embedding malware and phishing links within false content to compromise security systems [7]. As social platforms lack adequate verification mechanisms, unchecked misinformation undermines media credibility and threatens social stability and public trust. Therefore, early detection of false information and curbing its rampant spread is important and beneficial.

Fake information detection is a binary classification problem aimed at analyzing information content to evaluate its authenticity. Traditional approaches have focused on extracting semantic features, social media propagation patterns, and user interaction behaviors [8,9]. The advent of machine learning has introduced Recurrent Neural Networks (RNN) as a promising solution [10,11]. Despite their effectiveness in preliminary screening, these pre-trained single-modal detection models have inherent limitations in understanding the fundamental characteristics of misinformation. As multimedia technology develops, those disseminating false information use multimodal content, such as eye-catching images, to attract public attention. The change in tactics allows for the quicker and broader spread of misinformation, resulting in a more significant visual impact. Consequently, it can inflict greater harm on the target audience.

Detection methods have evolved to combine textual and visual features for improved accuracy [12,13]. Initial frameworks utilized LSTM and pre-trained VGG networks for feature extraction [14], though VGG19’s substantial computational requirements limit its efficiency [15]. Subsequent research advanced to BERT-based architectures with attention mechanisms to enhance cross-modal integration [16]. These approaches progressed from basic concatenation to sophisticated auxiliary tasks [17,18,19]. Recent advancements in co-attention networks facilitate effective modal interactions [20,21,22]. Several studies have explored the use of image–text alignment to optimize feature integration for fake news detection [2,23,24].

Multimodal false information shows a growing disparity in the distribution of features between textual and visual elements. Simple feature concatenation and unoptimized alignment approaches cannot effectively capture cross-modal semantic relationships and degrade fusion performance. Swarm intelligence optimization algorithms have emerged as an essential approach for feature optimization in multimodal detection [25]. The Beluga Whale Optimization (BWO) algorithm excels in global and local exploitation by simulating beluga whale behaviors, addressing high-dimensional nonlinear optimization tasks [26]. Nevertheless, BWO has limitations in convergence efficiency and feature adaptability for multimodal optimization [27]. Therefore, it is necessary to explore improvement strategies for the BWO algorithm.

We propose a false information detection model that integrates cross-modal attention-based feature fusion with an improved Beluga Whale Optimization algorithm to tackle these challenges. This research’s principal contributions are as follows:

1. We propose an Improved Beluga Whale Optimization (IBWO) algorithm to optimize textual and visual feature representations, enhancing feature adaptability before modal alignment.
2. We develop a supervised contrastive learning strategy for feature alignment, achieving semantic consistency between textual and visual modalities by maximizing the similarity of positive pairs while minimizing negative pairs.
3. We design a Cross-modal Attention Promotion (CAP) mechanism and global–local interaction learning pattern to capture dynamic modal relationships, generating high-quality fused feature representations.
4. We construct a multimodal false information detection dataset containing genuine and false text–image pairs to improve training effectiveness and experimental reliability.

## 2. Related Work

### 2.1. Unimodal Detection Method

In misinformation detection study, scholars have developed various single-modal approaches. These methods are fundamentally divided into text and visual feature detection strategies.

Text features: Early research in misinformation detection centers on analyzing textual content to evaluate authenticity. These methods rely primarily on statistical and semantic analysis, such as propagation time, structure, linguistic patterns [28,29], and emotional and stylistic elements [30]. As deceptive contents evolve, these features prove inadequate for identifying sophisticated false information [31]. The constraints of machine learning approaches prompt the ascent of deep learning-based solutions. Deep learning architectures enhance detection performance by extracting comprehensive features through underlying patterns and relationships. Ahmed et al. [32] introduced an innovative n-gram model that integrates text analysis, n-gram characteristics, and TF-IDF techniques and employs multiple machine learning classifiers for prediction. Drif et al. [33] proposed a framework integrating Convolutional Neural Networks (CNNs) for local feature extraction and Long Short-Term Memory (LSTM) networks for temporal dependency modeling. Furthermore, Mouratidis et al. [34] utilized paired text input patterns, enabling efficient and flexible fake information detection.

Visual features: The transition toward multimodal news content has prompted researchers to explore vision-based misinformation detection. These methods assess content authenticity by analyzing visual attributes, including color histograms, texture patterns, and shape characteristics. The comprehensive analysis of these visual properties enables effective discrimination between authentic and manipulated images. Jin et al. [35] proposed an approach based on visual clarity and diversity scoring, calculating image sets’ distribution differences and measuring visual variations within target news events to enhance detection accuracy. Elkasrawi et al. [36] combined reverse image search with edge detection and feature matching techniques to identify manipulated content. Qi et al. [37] developed a Multi-domain Visual Neural Network (MVNN) framework, employing CNN for frequency domain feature extraction and multi-branch CNN-RNN models for semantic analysis, with attention mechanisms dynamically fusing frequency and pixel domain features. Singh et al. [38] enhanced detection performance by integrating EfficientNet-B0 CNN with Error Level Analysis (ELA) to highlight image manipulation characteristics.

Although single-modal visual detection methods are effective, they have inherent limitations. Content manipulation risks and the lack of comprehensive validation cannot ensure information authenticity. These challenges have driven researchers toward multimodal approaches. Integrating visual and textual analysis enhances content evaluation, increasing detection accuracy and reliability.

### 2.2. Multimodal Detection Method

Multimodal feature fusion is crucial for fake news detection. Earlier studies [39,40,41] have focused on developing advanced feature extractors for various modalities and integrating them using simple concatenation methods. Although this straightforward approach achieves initial results, it shows limitations in the depth and accuracy of feature integration. Deep learning approaches for multimodal data have gained increasing attention in recent studies. Zhou et al. [42] designed a detection system utilizing visual and textual information similarity assessment and analyzed cross-modal relationships to identify fake news through inconsistencies between text and images. Xue et al. [43] designed the Multimodal Consistency Neural Network (MCNN) and employed five subnetworks to enhance detection accuracy through integrated text–image feature analysis. Subsequent studies have introduced more sophisticated integration methods. Qian et al. [44] created the Hierarchical Multimodal Context Attention Network (HMCAN), which combines multimodal information with hierarchical text semantics to enhance detection accuracy. Li et al. [45] proposed the Entity-oriented Multimodal Alignment and Fusion (EMAF) network focusing on entity relationships between images and text while maintaining semantic integrity. Recent developments have further refined these approaches. Song et al. [46] combined a Cross-modal Attention Residual Network with a Multi-channel Convolutional Network to achieve selective feature fusion while reducing noise interference. Zheng et al. [47] integrated textual, visual, and social structural features and considered modal complementarity and relationship alignments in social networks to modify detection effectiveness.

Multimodal feature fusion is essential for fake information detection, evolving from simple concatenation [39,40,41] to deep learning-based approaches [42,43,44,45] that capture cross-modal relationships. Recent advancements integrate attention mechanisms and structural alignments [46,47], enhancing detection accuracy and robustness.

### 2.3. Swarm Intelligence Optimization Algorithm

Swarm intelligence optimization algorithms have been widely applied to challenges in multimodal feature selection, fusion, and hyperparameter optimization [48,49,50]. These algorithms achieve global search and local optimization by simulating collective biological behaviors, offering strong adaptability and robustness. The Sparrow Search Algorithm (SSA) has demonstrated effectiveness in high-dimensional feature selection, significantly improving model classification performance [51]. However, when handling multimodal optimization, SSA often faces early convergence issues and struggles to balance exploration and exploitation. To address these challenges, Meng et al. [52] proposed an improved version of MSSSA by adding chaos mapping and opposition-based learning strategies. This improvement gains better global search capabilities and avoids local optima. Subsequently, Sun et al. [53] adapted SSA to binary form for more effective feature selection. Particle Swarm Optimization (PSO) has excelled in multimodal feature selection through its efficient global search and simple implementation [54,55]. Chen et al. [56] proposed Multi-Task PSO (MTPSO) to improve efficiency in high-dimensional optimization through task decomposition and knowledge-sharing mechanisms. Furthermore, Liu et al. [57] proposed a Random Particle Swarm Optimization (RPSO) algorithm based on cosine similarity, utilizing dynamic interactions to enhance search diversity and improve global optimization and classification performance. Other algorithms have also contributed significantly to feature selection methods. Grey Wolf Optimization (GWO) implements feature selection by modeling wolf-hunting behavior but tends to converge too early. Researchers have improved its performance through better population initialization and dynamic updates [58,59]. Similarly, the Firefly Algorithm and Zebra Optimization algorithm have achieved good results in feature selection and weight optimization [60,61,62,63]. In a comprehensive evaluation, Mu et al. [64] compared their BWO algorithm with 15 other optimization methods, demonstrating better performance in benchmark testing.

Current methods show limitations in capturing relationships across modalities and hierarchical semantic structures. Most detection methods only examine basic text features and miss the complete structural patterns [65]. Our fusion strategy leverages cross-modal attention networks to integrate feature- and decision-level information. By incorporating modal relationship learning and the enhanced Beluga Whale Optimization algorithm, our proposed model improves its ability to capture dynamic multimodal relationships, leading to higher detection accuracy.

## 3. Methods

**Figure 1:**
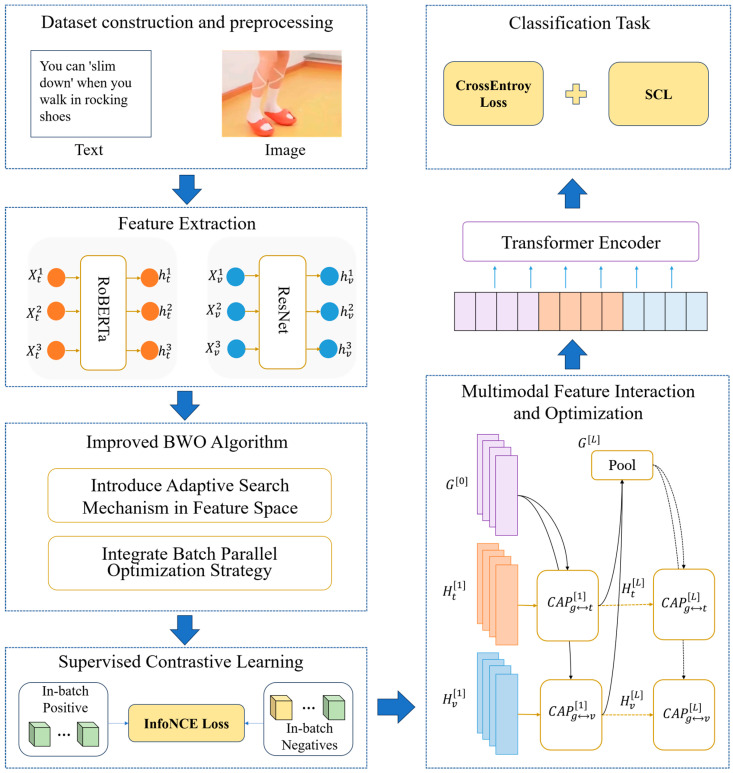
The architecture of the IBWO-CASC model.

This paper proposes a comprehensive framework for multimodal misinformation detection, effectively integrating textual and visual features. First, textual features are extracted with a pre-trained RoBERTa model, while visual features are obtained using the ResNet model. Then, an improved Beluga Whale Optimization (IBWO) algorithm enhances feature quality. Next, a supervised contrastive learning strategy is employed to align features from both modalities. Following alignment, an Attention mechanism is utilized to integrate the features. Finally, a Cross-modal Attention Promotion (CAP) mechanism and global–local interaction patterns merge features. Figure 1 presents the detailed framework.

### 3.1. Feature Extraction

#### 3.1.1. Text Encoding

The text modality is processed using the pre-trained RoBERTa model to abstract rich semantic features. Specifically, text sequences undergo basic preprocessing, including tokenization and stop-word removal, before converting into word vectors through an embedding layer. The RoBERTa model processes these vectors with [SOS] and [EOS] tokens marking sequence boundaries. The global text feature is obtained from the [EOS] token’s activation value in the model’s final layer. The text representations are further enhanced through layer normalization and linear transformation, projecting them into the multimodal embedding space. In addition, a masked self-attention mechanism in the encoder captures contextual relationships between words, enabling fine-grained semantic understanding. The formula is as follows.

(1) $$T_{i}=t_{1},t_{2},\ldots ,t_{n}$$

(2) $$H_{t}=\mathrm{RoBERTa}\left(T_{i}\right)$$

$T_{i}$ is the original input text, $t_{i}$ refers to the words or phrases that form the text, and n is the sequence length. The output $H_{t}=h_{t_{1}},h_{t_{2}},\ldots ,h_{t_{n}},h_{t_{cls}}$ represents the contextual features of the text, where $h_{t_{i}}$ is the contextual feature of the i-th word $t_{i}$ in the sequence.

#### 3.1.2. Image Coding

The image modality is mathematically defined as $V_{i}\in {\mathbb{R}}^{H\times W\times C}$, where H and W represent the height and width of the image, respectively, and C denotes the number of channels. This study employs the ResNet model to extract features from the imaging modality for robust feature representation. Before being fed into ResNet, input images are preprocessed through resizing and normalization to ensure consistency and stability in the data. ResNet’s unique residual connection mechanism effectively mitigates gradient vanishing and exploding gradients. The mechanism enables stable training in deep networks while preserving the flow of critical information.

For an input image $V_{i}$, ResNet produces a semantic representation $H_{i}=h_{i_{1}},h_{i_{2}},\ldots ,h_{i_{n}},h_{i_{cls}}$ as follows.

(3) $$H_{i}=\mathrm{ResNet}\left(V_{i}\right)$$

Here, $H_{i}$ represents the semantic features extracted from image $V_{i}$.

### 3.2. Proposal of the Improved Beluga Whale Optimization Algorithm

#### 3.2.1. Beluga Whale Optimization Algorithm

The Beluga whale optimization algorithm, proposed by Zhong et al. in 2022, is a heuristic method inspired by the natural behaviors of beluga whales [27]. The BWO algorithm involves exploration, development, and the whale drop phase. In the BWO algorithm, the balance factor $B_{f}$ and the whale fall probability $W_{f}$ are key parameters. They are critical for adjusting the algorithm’s global and local search abilities. Their definitions are as follows.

(4) $$B_{f}=r\left(1-\frac{T}{2Tmax}\right)$$

(5) $$W_{f}=0.1-\frac{0.05T}{Tmax}$$

In the equations, T signals the current iteration number, Tmax denotes the maximum number of iterations, and r is a random number within the range (0,1).

(1) In the exploration phase, the position of the beluga whale is updated according to Equation (6).

(6) $$\left\{ \begin{array}{c} X_{i,j}^{T+1}=X_{i,p_{j}}^{T}+X_{r,p_{1}}^{T}-X_{i,p_{j}}^{T}\times \left(1+r_{1}\right)\left(\sin2\pi r_{2}\right),j=even\\ X_{i,j}^{T+1}=X_{i,p_{j}}^{T}+X_{r,p_{1}}^{T}-X_{i,p_{j}}^{T}\times \left(1+r_{1}\right)(\cos2\pi r_{2}),j=odd \end{array}\right.$$

$X_{i,j}^{T+1}$ represents the new position of the i-th beluga whale in the j-th dimension. $p_{j}$ is a randomly selected integer from the d-dimensional space. $X_{i,p_{j}}^{T}$ denotes the position of the i-th beluga whale in the $p_{j}$-th dimension. $X_{r,p_{1}}^{T}$ and $X_{i,p_{j}}^{T}$ denote the current positions of a randomly selected beluga whale and the i-th beluga whale, respectively. $r_{1}$ and $r_{2}$ are random numbers within the range (0,1).

(2) The Lévy flight strategy is introduced in the exploitation phase. This phase is updated as follows:

(7) $$X_{i}^{T+1}=r_{3}\times X_{best}^{T}-r_{4}\times X_{i}^{T}+C_{1}\times L_{F}\times \left(X_{r}^{T}-X_{i}^{T}\right)$$

In the equation, $X_{i}^{T}$ and $X_{r}^{T}$ indicate the positions of the i-th beluga whale and a randomly selected beluga whale at iteration T, respectively. $X_{i}^{T+1}$ denotes the updated position of the i-th beluga whale, while $X_{best}^{T}$ refers to the best position found in the current population. $r_{3}$ and $r_{4}$ are random numbers in the range (0,1). The parameter $C_{1}$, which controls the strength of the Lévy flight jumps, is defined as $C_{1}=2r_{4}\times \left(1-T/Tmax\right)$. The Lévy flight function $L_{F}$ is as follows.

(8) $$L_{F}=0.05\times \frac{u\times \sigma }{|\nu {|}^{\frac{1}{\beta }}}$$

(9) $$\sigma ={\left(\frac{\Gamma \left(1+\beta \right)\times \sin(\frac{\pi \beta }{2})}{\Gamma \left(\frac{\left(1+\beta \right)}{2}\right)\times \beta \times 2^{\frac{\left(\beta -1\right)}{2}}}\right)}^{\frac{1}{\beta }}$$

In the equation, u and v are normal distributed random numbers; β is the default constant of 1.5.

(3) In the whale fall phase, the position is updated using the beluga whale’s current position and the whale fall’s step size. The specific formula is as follows.

(10) $$X_{i}^{T+I}=r_{5}X_{i}^{T}-r_{6}X_{r}^{T}+r_{7}X_{step}$$

In the equation, $r_{5}$, $r_{6}$, and $r_{7}$ are random numbers in the range (0,1). $X_{step}$ represents the whale fall step size, defined as $X_{step\;}=\left(ub-lb\right)\exp\left(-C_{2}\times \frac{T}{Tmax}\right)$. Here, $C_{2}$ is the step factor, calculated as $C_{2}=2W_{f}\times n$. ub and lb denote the upper and lower bounds of the variables, respectively.

#### 3.2.2. Improved Beluga Whale Optimization Algorithm (IBWO)

Based on the Beluga Whale Optimization (BWO) algorithm, this study proposes an improved method tailored for multimodal feature optimization. The proposed approach achieves efficient and stable results in high-dimensional multimodal feature spaces. In this study, the feature space refers to the high-dimensional representation of textual and visual data, where optimized features are projected for effective cross-modal alignment. The IBWO integrates an adaptive search mechanism and a batch parallel optimization strategy.

##### Introduced Adaptive Search Mechanism in Feature Space

The adaptive search mechanism in feature space is a dynamic optimization strategy designed to adjust search behavior according to the requirements of different optimization phases. During the early optimization stages, the algorithm focuses on global exploration, searching a broader solution space to identify potential high-quality solutions. As the iterations advance, the algorithm gradually shifts towards local exploitation, exploring more deeply into the solution space to refine and identify local optima. The following equation describes the search behavior during the optimization process.

(11) $$\vec{X}\left(t+1\right)=\{\begin{array}{cc} {\vec{X}}^{*}\left(t\right)-\vec{A}\cdot \vec{D}, & \mathrm{if}\;\left|\vec{A}\right|\geq 1\;(\mathrm{exploration})\\ {\vec{X}}^{*}\left(t\right)+\vec{A}\cdot \vec{D}, & \mathrm{if}\;\left|\vec{A}\right|<1\;(\mathrm{development}) \end{array}$$

Here, $\vec{X}\left(t+1\right)$ represents the feature position at the next iteration, while ${\vec{X}}^{*}\left(t\right)$ denotes the optimal feature position in the current population. $\vec{A}$ is the search coefficient that controls the direction and intensity of the search, defined as $\vec{A}=2aR_{1}-a$. $\vec{D}$ is the distance vector, representing the difference between the individual and the optimal position, calculated as $\vec{D}=\left|\vec{C}\cdot {\vec{X}}^{*}\left(t\right)-\vec{X}\left(t\right)\right|$. The convergence coefficient is defined as $\vec{C}=2R_{2}$.

When $\left|\vec{A}\right|\geq 1$, the algorithm performs global exploration, searching for potential optimization directions. Conversely, when $\left|\vec{A}\right|<1$, the algorithm focuses on local exploitation to accelerate convergence toward the optimal solution.

By adjusting the search coefficient $\vec{A}$ and the distance vector $\vec{D}$, this mechanism dynamically switches between global and local exploitation based on the optimization phase. This approach effectively prevents premature convergence while improving its efficiency. Additionally, the adaptive mechanism precisely guides the feature optimization process, enabling efficient and stable searches within high-dimensional and complex feature spaces.

##### Integrated Batch Parallel Optimization Strategy

The batch parallel optimization strategy is a mechanism that optimizes multiple sample features simultaneously, aiming to improve computational efficiency and reduce resource consumption. In this strategy, the algorithm processes a batch of sample features in parallel during each iteration, computes the objective function value for each sample, and updates the features concurrently. The objective function is defined as follows.

(12) $$L_{batch}=\frac{1}{B}\overset{B}{\underset{i=1}{\sum }}\;\overset{P}{\underset{j=1}{min}}{∥{\vec{X}}_{i}^{*}-{\vec{X}}_{ij}∥}_{2}^{2}$$

B represents the batch size, P denotes the population size, ${\vec{X}}_{i}^{*}$ is the target feature of the i-th sample, and ${\vec{X}}_{ij}$ refers to the j-th candidate feature of the i-th sample.

Through the batch parallel processing strategy, the algorithm can simultaneously optimize the feature vectors of multiple samples, significantly enhancing overall computational efficiency. Furthermore, high-dimensional feature optimization tasks can still be handled effectively under limited memory resources, maintaining the stability and scalability of the algorithm.

##### Adopted Early Stopping Mechanism

The early stopping mechanism is an optimization strategy designed to prevent unnecessary iterations, reduce computational costs, and improve training efficiency. Its core idea is to monitor changes in the objective function or evaluation metrics during optimization. The iteration process is terminated early to avoid resource wastage when the changes fall below a predefined threshold. Specifically, the iteration is terminated when the average fitness change $\Delta_{t}$ over p consecutive iterations satisfies the following condition.

(13) $$\Delta_{t}=\left|mean\left(F_{t}\right)-mean\left(F_{t-1}\right)\right|<\delta$$

p is the patience parameter with a default value of 5, while δ indicates the stopping threshold with a default value of $10^{-4}$.

#### 3.2.3. Multimodal Feature Optimization

In multimodal feature optimization, textual and image features exhibit distinct distribution characteristics. Multimodal features directly fusing may lead to inconsistencies between modalities and reduce the model’s detection performance. Therefore, this study utilizes the IBWO algorithm for multimodal tasks to optimize textual and image features, enhancing their representational capacity and improving compatibility between modalities.

The IBWO algorithm simulates the hunting behavior of beluga whales to dynamically adjust feature representations, allowing them to fit the training data distribution better. The optimized features are high-quality inputs that can replace the original features and significantly enhance the model’s performance during the fusion phase.

Assume that the textual features $X_{text}\in R^{B\times D}$ and image features $X_{image}\in R^{B\times D}$ represent the feature vectors extracted during the training phase. After dimensionality reduction, the two modalities are integrated into a unified feature representation $X\in R^{B\times D}$. The optimization objective of this study is to find the optimal feature representation in the feature space that minimizes the target function $f\left(X\right)$.

(14) $$\underset{X}{min}f\left(X\right),X\in R^{B\times D}$$

The target function $f\left(X\right)$ reflects the degree of fit between the features and the training data.

#### 3.2.4. The Steps of the IBWO Algorithm

Step 1: Define the objective function and set the relevant parameters for the IBWO algorithm.

Step 2: Initialize the parameters. Evaluate the initial fitness values for each individual and initialize the early stopping counter.

Step 3: Calculate the search coefficient for the current iteration according to Equation $\vec{A}=2aR_{1}-a$. When $\left|\vec{A}\right|\geq 1$, the algorithm performs global exploration. When $\left|\vec{A}\right|<1$, the algorithm focuses on local exploitation.

Step 4: Trigger the whale fall behavior with a certain probability and optimize the local solution according to Equation (11).

Step 5: In each iteration, calculate the fitness values of multiple individuals in parallel using Equation (12).

Step 6: Evaluate the current solution’s fitness. If it outperforms the global optimum, update the best solution and fitness value. If the fitness change is smaller than the threshold, stop optimization early as per Equation (13).

Step 7: Repeat Steps 3 to 6 until the maximum number of iterations is reached or the early stopping condition is satisfied.

### 3.3. Proposed Supervised Contrastive Learning for Feature Fusion

In multimodal fake news detection tasks, textual and image features often exhibit significant semantic differences. Directly merging these features can result in information loss or semantic inconsistencies, negatively impacting the model’s performance. Thus, this study proposes a supervised contrastive learning-based feature fusion strategy. This approach aligns textual and image features in the semantic space before fusion, ensuring consistency and improving the overall detection performance.

The key to this strategy lies in the feature alignment phase. By employing supervised contrastive learning (SCL), the model aligns the features of textual and image modalities in the embedding space. The alignment is achieved by maximizing the similarity of positive pairs (i.e., semantically related textual and image features) while minimizing the similarity of negative pairs (i.e., semantically unrelated textual and image features). The supervised contrastive learning loss function is defined as follows.

(15) $$L_{SCL}=-\frac{1}{N}\overset{N}{\underset{i=1}{\sum }}\log\left(\frac{e^{f\left(x_{i},x_{i}^{+}\right)}}{\sum_{j=1}^{K}e^{f\left(x_{i},x_{i_{j}^{-}}\right)}}\right)$$

N represents the number of samples in the batch. $x_{i}$ is the anchor sample, $x_{i}^{+}$ denotes the positive sample, and $x_{i_{j}^{-}}$ is the j-th negative sample. The function $f\left(x,y\right)$ is the mapping function of the representation learning model, which projects samples x and y into the latent space. $L_{SCL}$ represents the supervised contrastive learning loss.

The model computes similarity scores to align semantically related textual and image features in the representation space, minimizing the distance between positive pairs while maximizing that between negative pairs. This alignment ensures semantic consistency between modalities and provides a strong basis for effective feature fusion.

### 3.4. Deep Feature Fusion Using Attention Mechanisms

This paper employs an attention-based fusion method to integrate the aligned features fully. The approach utilizes the self-attention mechanism for intra-modal information interaction and the cross-attention mechanism for inter-modal information exchange.

**Figure 2:**
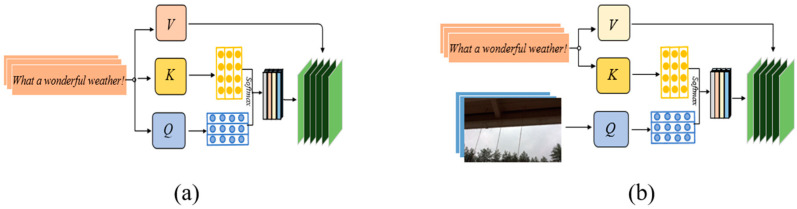
(**a**) Self-attention mechanism; (**b**) cross-attention mechanism.

The self-attention mechanism computes attention weights within a sequence, enabling the model to capture dependencies effectively. The multi-head self-attention mechanism extends this by employing multiple attention heads with distinct parameters to represent features from different subspaces. Taking the text modality as an example, we define the input to the multi-head self-attention mechanism as $H_{t}\in {\mathbb{R}}^{n\times d_{t}}$, where $d_{t}$ denotes the dimensionality of the text modality’s encoding. As illustrated in Figure 2a, the entire process can be represented as follows.

(16) $$Q_{i}=H_{t}W_{q}\in {\mathbb{R}}^{n\times d},\;K_{i}=H_{t}W_{k}\in {\mathbb{R}}^{n\times d},V_{i}=H_{t}W_{v}\in {\mathbb{R}}^{n\times d}$$

(17) $$head_{i}=\frac{softmax\left(Q_{i}K_{i}^{T}\right)}{\sqrt{d}}V_{i}$$

(18) $$MSA\left(H_{t}\right)=concat\left(head_{1},head_{2}\ldots ,head_{n}\right)W_{o}$$

$Q_{i}$, $K_{i}$, and $V_{i}$ represent the results of the linear transformations applied to the input vector by the i-th attention head. $W_{q}$, $W_{k}$, and $W_{v}$ are the weight matrices for the Query, Key, and Value mappings, respectively, which map the input to an output of d-dimensions. The operation concat denotes concatenation and $W_{o}$ denotes the final transformation’s weight matrix.

Figure 2b shows that the cross-attention mechanism manages dependencies across sequences by learning directional attention from the source to the target modality. Meanwhile, the cross-attention mechanism uses information from the source modality to enhance the target modality. Specifically, the query $Q_{j}$ is derived from the target modality $H_{t}$, while the key $K_{j}$ and value $V_{j}$ are obtained from the source modality $H_{v}$. The mappings are defined as $Q_{j}=H_{t}W_{q},K_{j}=H_{v}W_{k}$, and $V_{j}=H_{v}W_{v}$. This process facilitates interaction from modality $H_{t}$ to modality $H_{v}$.

(19) $$head_{j}=softmax\left(\alpha Q_{j}K_{j}^{T}\right)V_{j}$$

(20) $$MSA\left(H_{t},H_{v}\right)=concat\left(head_{1},head_{2}\ldots ,head_{n}\right)W_{o}$$

$H_{t}$ and $H_{v}$ represent different modalities and $MSA\left(H_{t},H_{v}\right)$ denotes the result of the multi-head cross-attention mechanism.

In multimodal misinformation detection tasks, the align-then-fuse strategy ensures semantic alignment between textual and image modalities. Self-attention and cross-attention mechanisms enhance feature interactions within and across modalities, ultimately generating fused feature representations with rich semantic expressiveness.

### 3.5. Innovative Multimodal Feature Interaction and Optimization Mechanism

#### 3.5.1. Proposed Cross-Modal Attention Promotion Mechanism (CAP)

To enhance inter-modal and intra-modal interactions, we propose an efficient Cross-Modality Attention Promotion mechanism (CAP). This mechanism employs symmetrical cross-modal attention to capture the intrinsic relationships between two input feature sequences. As a result, the CAP facilitates effective information exchange while enhancing each modality’s features during the fusion process.

The input features of each modality are first processed through a Feed-Forward Network (FFN) for linear transformation and refined using a nonlinear activation function, enhancing feature representation. Then, layer normalization (LN) is applied to ensure stability and proper numerical scaling. The CAP mechanism employs Multi-Head Self-Attention (MSA) to identify dependencies within each modality, strengthening key features and preparing them for inter-modal interaction. After intra-modal feature integration, CAP uses Multi-Head Cross-Attention (MCA) to enable interaction between modalities. In this process, text features serve as the query (Query), while image features act as the key (Key) and value (Value), and vice versa. This mechanism effectively captures semantic relationships between text and image modalities, producing fused cross-modal feature representations.

CAP takes two input sequences, $H_{t}$ and $H_{v}$, and outputs enhanced features $H_{t\to v}$ and $H_{v\to t}$. Using the *MCA*, the interaction between text and image modalities creates an intermediate feature representation $H_{t\to v}^{\prime }$.

(21) $$H_{t\to v}^{\prime }=MCA\left(LN\left(H_{t}\right),LN\left(H_{v}\right)\right)+H_{t}$$

Next, *MSA* further refines this intermediate representation to produce $H_{t\to v}^{\prime \prime }$.

(22) $$H_{t\to v}^{\prime \prime }=MSA\left(LN\left(H_{t\to v}^{\prime }\right)\right)+H_{t\to v}^{\prime }$$

Finally, *FFN* and *LN* are applied to generate the fused feature representation $H_{t\to v}$.

(23) $$H_{t\to v}=FFN\left(LN\left(H_{t\to v}^{\prime \prime }\right)\right)+H_{t\to v}^{\prime \prime }$$

This process ensures that the textual modality features are refined through multiple stages, achieving an optimal representational state before fusion. Similarly, the image modality features undergo the same refinement process, resulting in $CAP_{v↔t}\left(H_{t},H_{v}\right)$.

The CAP mechanism achieves mutual enhancement between modalities through information interaction and optimizes computational complexity. Considering that the computation time complexity of $MCA\left(H_{t},H_{v}\right)$ is $O\left(T_{t}T_{v}\right)$ and that of $MSA\left(H_{t}\right)$ is $O\left({T_{t}}^{2}\right)$, the total time complexity of one CAP is $O\left(T_{t}T_{v}+{T_{t}}^{2}+T_{t}T_{v}+{T_{v}}^{2}\right)=O\left({\left(T_{t}+T_{v}\right)}^{2}\right)$. Where $T_{t}$ denotes the sequence length of the text modality and $T_{v}$ signals the sequence length of the image modality.

#### 3.5.2. Proposed Global–Local Interaction Learning Framework

To further enhance the CAP mechanism’s effectiveness in multimodal feature fusion, this study introduces a Global–Local Interaction Learning Mode. In this context, global refers to cross-modal optimization, where a shared multimodal feature representation G is constructed to capture the macro relationships between text and images. On the other hand, local denotes independent optimization within each modality, where text and image features are modeled at a fine-grained level to preserve their respective informational characteristics. This mode optimizes the feature fusion process by facilitating interactions between global and local information, ensuring effective cross-modal information exchange while retaining the unique characteristics of each modality.

After the CAP mechanism generates the initial fused features, the sentence-level representations of each modality are treated as shared information and serve as the global multimodal context G, which interacts with local unimodal features. The process for creating the global multimodal context $G\left[i\right]$ is as follows.

(24) $$G\left[i\right]=concat\left(h_{t}^{i},h_{v}^{i}\right)\in R^{2\times d}$$

Here, i represents the layer number in the global–local interaction.

On the back of generating the global context $G\left[i\right]$, the model applies the MCA within the CAP framework to have local features $H_{t}^{i}$ and $H_{v}^{i}$ interact with the global multimodal context. This interaction integrates global information into the local features of each modality, ensuring that the final feature representation more effectively reflects the correlation and consistency between different modalities. The process is described as follows.

(25) $$H_{t}^{\left[i+1\right]},G_{t\to g}^{\left[i\right]}=CAP_{t↔g}^{\left[i\right]}\left(H_{t}^{\left[i\right]},G^{\left[i\right]}\right)$$

(26) $$H_{v}^{\left[i+1\right]},G_{i\to g}^{\left[i\right]}=CAP_{i↔g}^{\left[i\right]}\left(H_{v}^{\left[i\right]},G^{\left[i\right]}\right)$$

As the interaction progresses through multiple layers, the global context and local modality features are continuously refined and enhanced, forming richer and more abstract feature representations. After each interaction, the model aggregates information from different modalities using a pooling layer and further integrates it through a fully connected layer with Tanh-based nonlinear activation.

(27) $$G^{i+1}=softmax(v^{T}\tan h\left(W^{T}G_{g}^{T}+b\right))G_{g}$$

$G_{g}=concat\left(G_{v\to g}^{\left[i\right]},\;G_{t\to g}^{\left[i\right]}\right)$ represents the multimodal context information formed after global–local interaction.

Due to the small length of the global multimodal context, the overall time complexity is reduced to $O\left(\sum_{m=1}^{M}{\left(T_{m}+M\right)}^{2}\right)\approx O\left(\sum_{m=1}^{M}{T_{m}}^{2}\right)$, as in practice, $M\;\ll \;T_{m}$. Under modality alignment, it further simplifies to $O\left(MT^{2}\right)$. Therefore, the global–local fusion strategy of CAP achieves linear complexity in terms of space and linear computation efficiency.

### 3.6. Classification Task

A multi-task learning framework is designed to improve classification performance for multimodal fake information detection. This framework incorporates cross-entropy and supervised contrastive loss to handle information from different modalities effectively.

The prediction results for the text modality, image modality, and their combined modality are calculated separately and fused to generate the final prediction value. The following formula represents this process.

(28) $$\hat{y}=y_{t}+y_{i}+y_{f}$$

$y_{t}$, $y_{i}$, and $y_{f}$ represent the prediction values for the text modality, image modality, and their fusion, respectively.

The objective of the cross-entropy loss function is to minimize the difference between the predicted values and the true labels. It is defined as follows.

(29) $$L_{pred}=\overset{N}{\underset{i\in \left\{t,v,f\right\}}{\sum }}\left[y^{i}\log{\hat{y}}^{i}+\left(1-y^{i}\right)\log\left(1-{\hat{y}}^{i}\right)\right]$$

L indicates the loss function for classification, y is the actual label of the sample (0 for real information and 1 for fake information), and ${\hat{y}}^{i}$ signifies the predicted result of the sample by the model.

As previously detailed, the SCL loss is employed to optimize multimodal feature alignment. To further improve the performance of the classification task, the SCL loss is combined with the cross-entropy loss. The final optimization objective function is as follows.

(30) $$L=L_{pred}+\alpha L_{SCL}$$

α denotes the hyperparameter adjusting the loss function weights.

## 4. Experiment Analysis

### 4.1. Data Collection and Preprocessing

Datasets are collected from CCTV.com and several rumor-debunking platforms to verify the proposed model’s effectiveness. CCTV.com is an authoritative central news website operated by the China Media Group. Accurate information comes from CCTV.com, focusing on officially verified content published between April 2022 and November 2023. Duplicate data caused by media outlets posting identical text or images across multiple accounts are removed by comparing text and image files. Irrelevant or meaningless content are also filtered out, resulting in a cleaned dataset of 2817 text–image pairs.

Fake information is gathered from platforms such as the China Internet Joint Rumor Debunking Platform, Science Rumor Debunking, Science China, and Tadpole Pentatonic. Data from August 2019 to December 2023 are used in the same preprocessing steps. After filtering, 3605 valid entries are retained from an initial 4525 samples. Real information is labeled positive, while fake information is labeled negative in the final dataset. Each sample contains text paired with its associated image.

The dataset is split in a ratio of 8:1:1 for training, validation, and testing, respectively. We use undersampling and oversampling techniques to maintain a balanced distribution of positive and negative samples.

### 4.2. Experimental Setup and Evaluation Metrics

The hyperparameter settings for the base model are shown in Table 1.

Fake information detection is a classification problem. Therefore, metrics such as accuracy, precision, recall, and *F*1-score are commonly used to evaluate the results.

(31) $$Accuracy=\frac{TP+TN}{TP+FN+FP+TN}$$

(32) $$Precision=\frac{TP}{TP+FP}$$

(33) $$Recall=\frac{TP}{TP+FN}$$

(34) $$F1=\frac{2*Precision*Recall}{Precision+Recall}$$

### 4.3. Comparative Experiments

Several comparative experiments are conducted to evaluate the performance of the proposed IBWO-CASC model. These experiments include single-modality models, multimodal fusion models, models incorporating the improved optimization algorithm, and comparisons with advanced baseline models. Additionally, all models are run under identical environments to ensure the robustness of the experimental results. Moreover, cross-validation is employed to minimize the impact of random factors.

#### 4.3.1. Analysis of Single-Modality Experiment Results

Experiments are conducted on the constructed dataset to determine the most appropriate architecture for processing single-modality data. For text classification, models such as TextCNN, BiLSTM, SMSD, CLIP-Text, and RoBERTa are selected, representing different feature extraction and sequence modeling approaches. CLIP-Image and ResNet are used for image classification for their strong visual feature extraction capabilities. The results are shown in Table 2.

The results indicate that traditional models do not perform as well as expected. TextCNN achieves an accuracy of 82.77%, while BiLSTM shows an increase of 0.32%. TextCNN is effective for short-text processing by relying on convolutional operations to extract local features. However, TextCNN struggles with global semantic modeling for longer texts. BiLSTM utilizes bidirectional modeling to capture contextual dependencies better, but it is still limited in handling complex semantics. SMSD achieves an accuracy of 0.26% higher than TextCNN but 0.06% lower than BiLSTM. Despite using a self-matching mechanism to measure text similarity, SMSD’s semantic modeling remains shallow and reduces its ability to capture deep semantic features in fake news detection. Compared to traditional models, pre-trained language models indicate significant performance improvements. BERT demonstrates an accuracy 4.71% higher than BiLSTM, showcasing its superior ability to model global semantic relationships using a transformer-based architecture. CLIP-Text further improves on BERT, with an accuracy increase of 0.34%, highlighting its strong semantic modeling capabilities. RoBERTa, an optimized version of BERT, delivers the best results across all metrics. Its accuracy exceeds CLIP-Text by 1.69%, making it this experiment’s most effective text classification model. RoBERTa’s improved training strategies and advanced deep semantic modeling capabilities underscore its advantages in fake news detection tasks.

For image classification, ResNet achieves an accuracy of 85.66%, demonstrating strong capabilities in visual feature extraction and suitability for single-modality tasks. CLIP-Image’s accuracy is 1.09% lower than ResNet’s, indicating weaker feature extraction capabilities for single-modality image tasks.

Although single-modality models perform reasonably well within their respective domains, they cannot utilize complementary information from other modalities, ultimately limiting their effectiveness.

#### 4.3.2. Analysis of Multimodal Experiment Results

The multimodal fusion models employ different strategies to integrate text and image modalities. After extraction, the C model performs initial fusion by simply concatenating text and image features. Based on the C model, the CA model incorporates an Attention mechanism to enhance interactions between the two modalities. The CAS model further improves semantic alignment by introducing Supervised Contrastive Learning (SCL). The CAC model strengthens cross-modal information exchange using the Cross-Modality Attention Promotion mechanism (CAP). Finally, the CASC model combines SCL and CAP to achieve efficient alignment and deep integration of multimodal features. Table 3 presents the multimodal fusion model results, with the best results highlighted in bold.

The experimental results demonstrate that multimodal fusion strategies significantly enhance fake news detection performance, improving the model’s effectiveness as fusion methods are optimized. The C model achieves initial fusion through simple feature concatenation, boosting accuracy by 3.75% compared to the best single-modal model, RoBERTa. However, its performance gain is limited due to the lack of inter-modal relationship modeling. The CA model integrates an Attention mechanism, raising accuracy to 94.23% and validating the effectiveness of deep interaction mechanisms in modality integration. The CAS model further incorporates Supervised Contrastive Learning (SCL), optimizing modality alignment and improving accuracy by 1.91% over the CA model. While the CAC model introduces the Cross-Modality Attention Promotion (CAP) mechanism, achieving slightly lower accuracy than the CAS model, it demonstrates superior robustness and stability in high-noise scenarios. Finally, the CASC model combines the strengths of SCL and CAP and outperforms all other models. The CASC model underscores the importance of modality alignment and deep interaction in multimodal fake news detection.

#### 4.3.3. Comparative Analysis of Swarm Intelligence Optimization Algorithms

The PSO-CASC model integrates the Particle Swarm Optimization (PSO) algorithm with the CASC framework. PSO is widely recognized as a classical swarm intelligence optimization method, particularly excelling in high-dimensional optimization and feature selection tasks. Compared to BWO and IBWO, PSO is easier to implement, computationally more efficient, and provides strong global search capabilities with faster convergence. Thus, PSO is used as a baseline algorithm in this study to validate the advantages of BWO and IBWO for solving complex optimization tasks. The BWO-CASC and IBWO-CASC models incorporate the Beluga Whale Optimization (BWO) and Improved Beluga Whale Optimization (IBWO) algorithms into the CASC framework. Table 4 shows the results, with the best results highlighted in bold.

The PSO-CASC model integrates the Particle Swarm Optimization (PSO) algorithm into the CASC framework, yielding slight performance gains. Compared to the CASC model, accuracy, precision, recall, and F1 scores increase by 0.07%, 0.29%, 0.16%, and 0.19%, respectively. These results highlight PSO’s ability to enhance global search and local exploitation for effective feature selection. However, the relatively minor improvements indicate the algorithm’s limitations in fully capturing the complexity of high-dimensional feature interactions. The BWO-CASC model incorporates the Beluga Whale Optimization (BWO) algorithm, achieving an accuracy of 0.07% higher than PSO-CASC. By simulating the hunting behavior of beluga whales, BWO improves global search capabilities and parameter tuning. Nevertheless, the advancements remain incremental, reflecting the inherent constraints of BWO in managing complex multimodal feature spaces and achieving more profound optimization. The IBWO-CASC model shows a significant performance improvement, surpassing the BWO-CASC, with gains of 1.05%, 1.12%, 0.98%, and 1.04% in accuracy, precision, recall, and F1 score, respectively. These advances are attributed to IBWO’s enhanced dynamic balancing mechanism, which optimizes global exploration and local exploitation more effectively. This mechanism enables the model to adapt to high-dimensional feature spaces, aligning multimodal features with greater precision while minimizing noise and redundancy. As a result, IBWO-CASC achieves more profound feature fusion and more robust predictions. Compared to the CASC model, IBWO-CASC achieves an accuracy improvement of 1.19%, underscoring the significant impact of the enhanced IBWO algorithm. While PSO and BWO have notable development, the IBWO model introduces notable improvements in addressing the challenges of multimodal feature alignment and fusion.

#### 4.3.4. Comparative Analysis of Baseline Models

The proposed model is evaluated against six baseline models using the constructed dataset. The experiment results are summarized in Table 5, with the best-performing results highlighted in bold.

The experimental results show that the IBWO-CASC model improves accuracy by 9.29% over EANN, 5.51% over SAFE, 2.55% over CSFND, 2.42% over FSRU, 2.45% over CLKD-IMRD, and 2.70% over HSEN. The IBWO-CASC model surpasses six baseline models in detecting multimodal fake news, demonstrating superior accuracy and efficiency. This model shows promise for advancing research and practical applications in this area.

EANN [18]: This model uses an event adversarial mechanism to enhance robustness and improve multimodal fake news detection. However, its inability to fully utilize multimodal features limits its performance.

SAFE [42]: It detects mismatches between cross-modal features by analyzing text–image relationships. While it improves detection accuracy, its restricted understanding of complex feature interactions constrains its effectiveness.

CSFND [66]: The model mitigates inconsistencies between semantic and decision spaces through contextual semantic representation learning. However, it struggles to integrate cross-modal features deeply, limiting adaptability.

FSRU [67]: It relies on Fourier transforms to extract spectral features and combines dual contrastive learning for multimodal fusion. Although the model is strong in spectral analysis, it lacks effectiveness in non-spectral feature integration and complex multimodal interactions.

CLKD-IMRD [68]: Semantic coherence between text and images is strengthened through contrastive learning and knowledge distillation is employed to solve missing modalities. While it performs well in certain situations, it struggles with modeling global relationships and adapting to real-time detection needs.

HSEN [69]: It generates image descriptions aligned with text via reinforcement learning and uses adaptive attention mechanisms to filter out noise, thereby enhancing reliability. However, its limited capacity for global interactions and deeper multimodal integration restricts its overall performance.

IBWO-CASC (Ours): The model dynamically optimizes high-dimensional text and image features using the Improved Beluga Whale Optimization (IBWO) algorithm, boosting feature selection efficiency. It achieves precise semantic alignment through supervised contrastive learning (SCL), reducing noise and redundancy. The Cross-Modality Attention Promotion (CAP) mechanism enhances dynamic intra-modal and cross-modal interactions, ensuring superior feature fusion. Our proposed model outperforms all baseline models in accuracy, recall, and F1 score, demonstrating exceptional adaptability and robustness.

#### 4.3.5. Comparative Analysis of Model Complexity and Computational Efficiency

Table 6 presents a comparative analysis of model complexity, evaluating the parameter count (Params) and computational cost (FLOPS) across different models. These two metrics are essential for understanding the trade-off between model accuracy and computational efficiency. Params represent the total number of trainable parameters in each model, measured in millions (M). A higher parameter count generally enhances learning capacity and increases memory consumption and computational demands. FLOPS measures the number of floating-point operations required for a single forward pass, expressed in billions (G). Higher FLOPS indicate greater computational cost, which can impact inference speed and deployment feasibility, particularly in real-time applications.

IBWO-CASC has 742.15 M parameters, making the model relatively lightweight compared to CSFND and FSRU. The reduced number of parameters improves efficiency in computational complexity and storage, which is beneficial for practical applications. In terms of FLOPS, IBWO-CASC records 37.89 G, significantly lower than SAFE and CSFND, demonstrating a clear advantage in resource consumption. Although the FLOPS count is slightly higher than that of EANN and ResNet, the optimized feature fusion strategy enhances accuracy, maintaining a balance between efficiency and performance.

**Figure 3:**
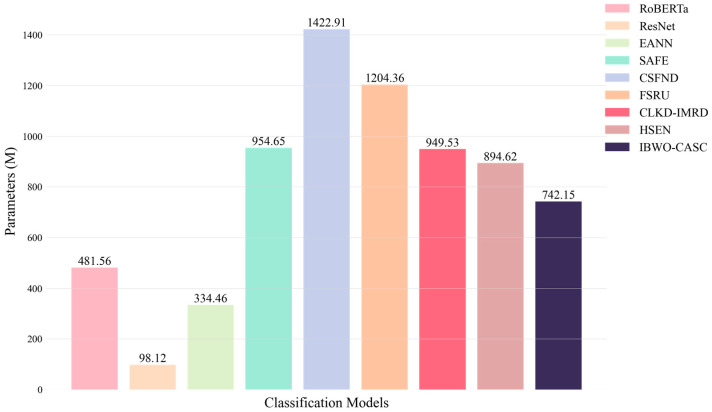
Comparison of Models’ Parameters.

**Figure 4:**
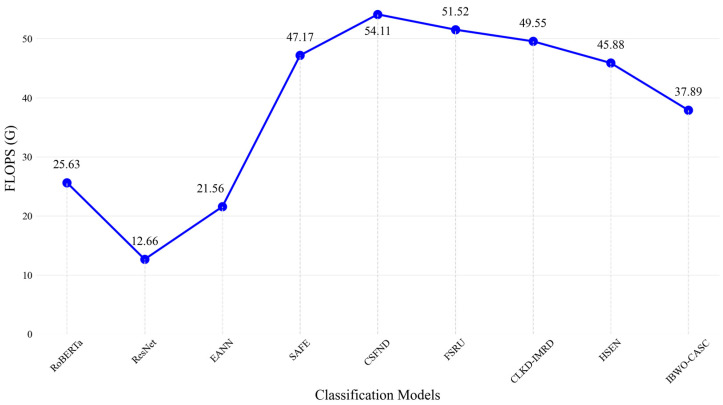
Comparison of Models’ FLOPS.

As shown in Figure 3 and Figure 4, the bar and line charts provide a clear comparison of model parameters and FLOPS, demonstrating that IBWO-CASC maintains high performance while optimizing computational efficiency.

### 4.4. Visualization Analysis

We present a visualization analysis focusing on training convergence and feature representation learning better to understand the model’s performance and feature representation capabilities.

**Figure 5:**
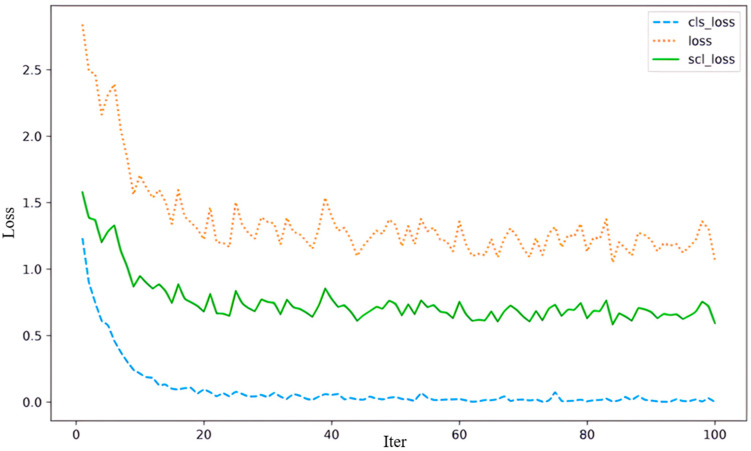
Loss function convergence curves.

Firstly, Figure 5 illustrates the convergence trends of different loss functions during model training, including total loss (loss), classification loss (cls_loss), and contrastive loss (scl_loss). As training progresses, all three loss values steadily decrease and eventually stabilize. The rapid decline in classification loss during the early stages highlights the model’s ability to effectively learn essential features for the classification task. Meanwhile, the steady decline in contrastive loss demonstrates the success of the contrastive learning strategy in aligning multimodal features and enhancing feature fusion. The smooth convergence of these loss functions reflects the stability of the training process and validates the proposed method’s efficiency and robustness in optimization.

**Figure 6:**
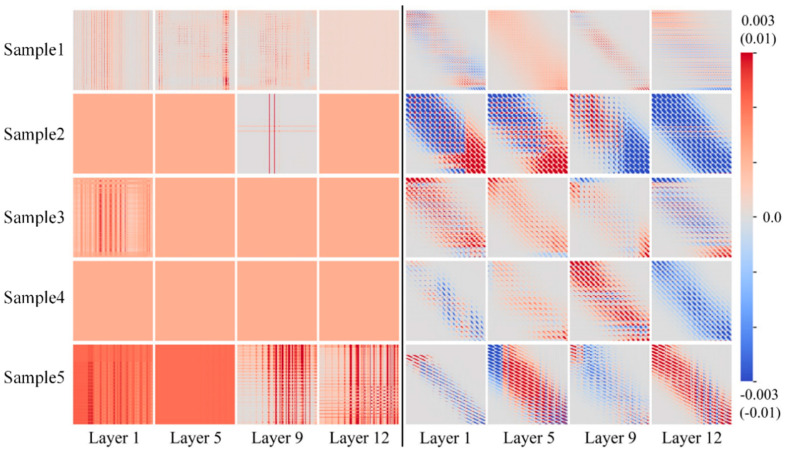
Multi-layer attention feature heatmaps.

Secondly, Figure 6 illustrates attention heatmaps across different layers, highlighting the model’s focus on various feature levels. In lower Layer 1, attention primarily targets local features, capturing detailed information from text and images. In higher Layer 12, attention shifts towards global feature fusion and interaction. The comparison shows that attention distributions at higher layers become more refined and precise, demonstrating the model’s capability to capture fine-grained information at deeper levels. This behavior aligns with the proposed multimodal fusion approach, which leverages deep feature interactions to improve classification performance. Additionally, it complements the cross-modal fusion strategy, showcasing the model’s advantage in multi-layer feature modeling.

The two figures highlight our approach’s strengths in the training process and hierarchical feature learning. They validate that the proposed multimodal fusion strategy improves classification accuracy and facilitates deep-integration and representation of cross-modal information through effective contrastive learning and multi-layer attention mechanisms, significantly boosting the model’s overall performance.

## 5. Conclusions and Prospects

### 5.1. Conclusions

This paper proposes the IBWO-CASC model, a multimodal fake news detection framework integrating the improved Beluga Whale Optimization (IBWO) algorithm and the Cross-Modality Attention Promotion (CAP) mechanism. This model addresses the challenges of feature alignment and fusion in fake information detection.

Firstly, the IBWO algorithm proves essential in optimizing feature representations and enhancing overall model performance. By balancing global exploration and local exploitation, IBWO overcomes the limitations of traditional optimization algorithms in convergence speed and feature adaptability. Experimental results demonstrate that IBWO-CASC shows enhancements of 1.19%, 1.44%, 1.19%, and 1.25% in accuracy, precision, recall, and F1 score, respectively, compared to the CASC model without optimization. These results underscore the effectiveness of IBWO in optimizing modality alignment and feature fusion in high-dimensional spaces.

Secondly, the supervised contrastive learning (SCL) mechanism significantly improves semantic alignment between text and image modalities. By maximizing positive pairs’ similarity and minimizing that of negative pairs, SCL reduces noise and redundancy across modalities. Incorporating SCL into the CAS model boosts accuracy by 4.31% compared to the baseline C model, showcasing its strong capability in modality alignment.

Thirdly, the CAP mechanism further enhances feature fusion depth and quality by capturing dynamic intra-modal and cross-modal interactions. With a global–local interaction learning strategy, CAP modifies information exchange between modalities and enhances robustness in complex scenarios. Experimental results indicate that the CAC model with CAP achieves a 1.52% accuracy improvement over the baseline CA model, demonstrating its high stability and adaptability.

Finally, compared to other swarm intelligence algorithms and baseline models, IBWO-CASC consistently outperforms in multiple evaluation metrics. While the average accuracy of baseline models was 93.58%, IBWO-CASC achieves a 4.09% advancement. Furthermore, its optimization process is more efficient, reducing computational resource requirements while maintaining superior performance in multimodal tasks.

In summary, the IBWO-CASC model combines the IBWO algorithm, SCL mechanism, and CAP mechanism to achieve efficient alignment and deep fusion of text and image modalities, demonstrating outstanding performance in multimodal fake information detection tasks.

### 5.2. Future Prospect

Although our proposed model achieves high accuracy in false information detection, future research offers several avenues for improvement. First, the model should incorporate more complex modalities, such as video and audio, to improve feature alignment and fusion. Second, optimizing computational efficiency to develop lightweight versions for resource-constrained environments should increase its applicability in real-time detection tasks. Finally, integrating explainability techniques should strengthen user trust and contribute to creating a more transparent and sustainable online ecosystem.

## Figures and Tables

**Table 1:** Model Hyperparameter Settings.

Hyperparameters	Value
Text Embed Dim	512
Image Embed Dim	512
Lr	3 × 10^−4^
Layers	12
Attention Heads	8
Dropout	0.3

**Table 2:** Comparison Results of Single-Modality Models.

Module	Model	Accuracy	Precision	Recall	F1
T	TextCNN	0.8277	0.8194	0.8203	0.8204
T	SMSD	0.8299	0.8177	0.8184	0.8188
T	Bi-LSTM	0.8304	0.8291	0.8298	0.8301
T	BERT	0.8695	0.8688	0.8685	0.8686
T	Clip-Text	0.8725	0.8696	0.8699	0.8698
T	RoBERTa	0.8873	0.8866	0.8861	0.8863
I	Clip-Image	0.8473	0.8451	0.8456	0.8455
I	ResNet	0.8566	0.8498	0.8495	0.8496

**Table 3:** Comparison Results of Multimodal Models.

Module	Model	Accuracy	Precision	Recall	F1
T+I	C	0.9206	0.9199	0.9198	0.9198
T+I	CA	0.9423	0.9419	0.9418	0.9418
T+I	CAS	0.9603	0.9593	0.9586	0.9591
T+I	CAC	0.9566	0.9512	0.9514	0.9512
**T+I**	**CASC**	**0.9626**	**0.9613**	**0.9612**	**0.9617**

**Table 4:** Comparative Experiments of Swarm Intelligence Optimization Algorithms.

Module	Model	Accuracy	Precision	Recall	F1
T+I	CASC	0.9626	0.9613	0.9612	0.9617
T+I	PSO-CASC	0.9633	0.9641	0.9628	0.9635
T+I	BWO-CASC	0.9640	0.9643	0.9632	0.9637
**T+I**	**IBWO-CASC**	**0.9741**	**0.9751**	**0.9726**	**0.9737**

**Table 5:** Comparative Experiments of Baseline Models.

Module	Model	Accuracy	Precision	Recall	F1
T+I	EANN	0.8913	0.8807	0.8861	0.8816
T+I	SAFE	0.9232	0.9187	0.9107	0.9151
T+I	CSFND	0.9499	0.9468	0.9472	0.9472
T+I	FSRU	0.9511	0.9505	0.9512	0.9511
T+I	CLKD-IMRD	0.9508	0.9499	0.9504	0.9502
T+I	HSEN	0.9485	0.9458	0.9449	0.9456
**T+I**	**IBWO-CASC**	**0.9741**	**0.9751**	**0.9726**	**0.9737**

**Table 6:** Comparative Experiments of Model Complexity.

Model	Params	FLOPS
RoBERTa	481.56 M	25.63 G
ResNet	98.12 M	12.66 G
EANN	334.46 M	21.56 G
SAFE	954.65 M	47.17 G
CSFND	1422.91 M	54.11 G
FSRU	1204.36 M	51.52 G
CLKD-IMRD	949.53 M	49.55 G
HSEN	894.62 M	45.88 G
IBWO-CASC	742.15 M	37.89 G

## Data Availability

The raw data and code supporting the conclusions of this article will be made available by the authors on request.
